# An Attention-Residual Convolutional Network for Real-Time Seizure Classification on Edge Devices

**DOI:** 10.3390/s25226855

**Published:** 2025-11-10

**Authors:** Peter A. Akor, Godwin Enemali, Usman Muhammad, Rajiv Ranjan Singh, Hadi Larijani

**Affiliations:** School of Science and Engineering, Glasgow Caledonian University, Glasgow G4 0BA, UK; peter.akor@gcu.ac.uk (P.A.A.); godwin.enemali@gcu.ac.uk (G.E.); muhammad.usman@gcu.ac.uk (U.M.); rajiv.singh@gcu.ac.uk (R.R.S.)

**Keywords:** seizure classification, attention mechanism, residual network, edge computing, epilepsy monitoring, Raspberry Pi, EEG analysis

## Abstract

Epilepsy affects over 50 million people globally, with accurate seizure type classification directly influencing treatment selection as different seizure types respond to specific antiepileptic medications. Manual electroencephalogram (EEG) interpretation remains time-intensive and requires specialized expertise, creating clinical workflow bottlenecks. This work presents EEG-ARCNet, an attention-residual convolutional network integrating residual connections with channel attention mechanisms to extract discriminative temporal and spectral features from multi-channel EEG recordings. The model combines nine statistical temporal features with five frequency-band power measures through Welch’s spectral decomposition, processed through attention-enhanced convolutional pathways. Evaluated on the Temple University Hospital Seizure Corpus, EEG-ARCNet achieved 99.65% accuracy with 99.59% macro-averaged F1-score across five seizure types (absence, focal non-specific, simple partial, tonic-clonic, and tonic). To validate practical deployment, the model was implemented on Raspberry Pi 4, achieving a 2.06 ms average inference time per 10 s segment with 35.4% CPU utilization and 499.4 MB memory consumption. The combination of high classification accuracy and efficient edge deployment demonstrates technical feasibility for resource-constrained seizure-monitoring applications.

## 1. Introduction

Epilepsy is a chronic neurological disorder characterized by recurrent seizures from sudden abnormal electrical activity in the brain [1]. This condition affects approximately 50 million people worldwide, with nearly 80% residing in low- and middle-income countries [2]. As the second most common neurological disorder after stroke [3], epilepsy creates substantial healthcare system burdens globally [4]. Epilepsy patients face mortality rates 2–3× higher than those of the general population [5], emphasizing the importance of timely and accurate diagnosis.

Appropriate antiepileptic medication selection depends fundamentally on accurate seizure type identification as different seizures respond to specific therapeutic agents. For focal seizures, medications such as carbamazepine, oxcarbazepine, and lamotrigine serve as first-line treatments [6]. Generalized seizures require different approaches: valproate functions as a broad-spectrum medication effective for absence and myoclonic seizures, while ethosuximide has a narrower indication limited primarily to absence seizures [7]. This precision becomes particularly critical for patients who develop drug-resistant epilepsy showing limited response to standard treatments such as primidone and phenytoin [8]. When medications fail, surgical intervention may be considered, where accurate seizure classification directly influences treatment success [9].

Clinical diagnosis traditionally relies on electroencephalogram (EEG) recordings, but interpreting these signals demands considerable time and specialized neurological expertise [10]. This requirement creates significant epilepsy care disparities. Many patients experience prolonged diagnostic delays or lack access to qualified specialists [11,12]. When standard EEG evaluations prove insufficient, clinicians turn to video–EEG monitoring (VEM), which combines EEG with synchronized video recordings. While more comprehensive, this approach requires lengthy hospital stays and intensive manual review by trained neurologists [10], making it resource-intensive and less accessible.

These challenges have motivated researchers to explore automated classification systems using machine learning. Early deep learning approaches showed varying success. Transfer learning with InceptionV3 achieved 88.3% accuracy [13], while CNN-LSTM [14] and neural memory network [15] architectures reached F1-scores of 97.4% and 94.5%, respectively. These systems, however, often performed well only under controlled conditions and struggled in diverse clinical scenarios.

Two main research directions emerged: feature-based methods and end-to-end deep learning [16]. Feature-based approaches showed consistent performance across different EEG applications [3,17,18,19]. Time-frequency analysis techniques, particularly wavelet transforms combined with statistical methods, became widely adopted [3,17,18,20,21] for capturing both temporal dynamics and spectral characteristics. End-to-end methods, while theoretically capable of learning hierarchical representations automatically, often face practical challenges with computational efficiency and clinical reliability.

Recent work has incorporated attention mechanisms to improve feature learning. Baghdadi et al. [22] developed a channel-wise attention LSTM model, achieving a 98.41% F1-score, demonstrating how attention helps networks focus on diagnostically relevant signal components. Albaqami et al.’s MP-SeizNet [23] employed a multi-path architecture combining CNNs and bidirectional LSTMs. Multi-representation learning, which processes EEG data through different analytical pathways, has emerged as a promising innovation [24,25,26], allowing models to capture complementary seizure activity aspects.

Recent advances in EEG-based brain–computer interfaces have explored sophisticated neural architectures beyond seizure classification. Hu et al. [27] developed STRFLNet for emotion recognition, combining spatial graph convolution with temporal self-attention to capture complex spatio-temporal dependencies. Their multi-scale fusion approach integrates spatial and temporal representations’ value, although with substantial computational requirements (approximately 180 MFLOPs). Cai et al. [28] proposed EEG-SWTNS, converting EEG signals to spectral images through short-time wavelet transform before neural network processing, achieving strong emotion recognition through visualization-based approaches. While these methods have advanced EEG-based BCI research, they have typically prioritized classification accuracy over computational efficiency, limiting deployment on resource-constrained devices. Recent transformer-based approaches have also explored self-attention for EEG analysis, with vision transformer adaptations achieving strong performance but requiring substantial computational resources (>200 MFLOPs), limiting edge deployment [29]. Lightweight architectures adapted from computer vision, such as MobileNetV3 variants for EEG, demonstrate efficient inference but often sacrifice accuracy for speed [30]. Our work bridges this gap by balancing high accuracy with edge deployment feasibility for clinical seizure monitoring.

Our previous work [31] addressed seizure classification using cascaded architecture with two specialized CNNs—a binary detector followed by multi-class classifier—achieving over 99% accuracy across seven seizure types by decomposing the classification problem to handle extreme class imbalance. While that hierarchical approach proved effective, it required two separate models with independent training pipelines and was not evaluated for deployment on resource-constrained devices.

A critical consideration for real-world deployment involves computational requirements. Many current architectures demand substantial resources, limiting practical applicability. Advanced attention-based methods often require parallel processing across multiple neural networks. For example, channel-wise approaches process each EEG channel through separate encoders, with some implementations using approximately 29,000 parameters per channel encoder, totaling over 10 million parameters [32]. Such architectures typically require GPU acceleration and multi-stage training due to hardware constraints, with inference times around 15 ms even with GPU support. Similarly, multi-path architectures combining CNNs with bidirectional LSTMs in parallel processing streams add considerable computational overhead [23]. These demands create barriers to deployment in resource-limited settings and continuous monitoring scenarios where energy efficiency matters.

Edge computing offers a solution to these constraints. By processing data locally on devices rather than relying on cloud infrastructure, edge computing reduces latency, addresses privacy concerns, and enables operation in environments with limited connectivity [33]. For epilepsy monitoring, this means seizure detection algorithms could run directly on portable devices, providing immediate feedback without requiring constant connection to external servers. Recent implementations of edge computing for healthcare have demonstrated that carefully designed models can achieve clinically useful performance while operating within low-power device constraints [34]. However, few studies have validated seizure classification systems on actual edge hardware with comprehensive performance metrics.

This work presents EEG-ARCNet, which addresses both accuracy and practical deployment. Unlike channel-wise attention methods or multi-path architectures, EEG-ARCNet integrates attention within residual blocks, enabling efficient computation while maintaining high performance. The architecture processes five seizure types (absence, focal non-specific, simple partial, tonic–clonic, and tonic) using 758,725 parameters. We extract nine temporal statistical measures and five frequency-band power features through Welch’s spectral decomposition, processed through attention-enhanced convolutional layers with residual connections. We validated deployment on Raspberry Pi 4B (Raspberry Pi Foundation, Cambridge, United Kingdom) with comprehensive performance evaluation.

The key contributions are as follows:

- A novel attention-residual convolutional network. achieving 99.65% accuracy with 99.59% F1-score while requiring substantially fewer computational resources than existing attention-based approaches.
- Efficient channel attention integrated with dual-domain feature extraction, representing significant complexity reduction compared to multi-stage hierarchical and channel-wise methods.
- Successful edge deployment on Raspberry Pi (Raspberry Pi Foundation, Cambridge, United Kingdom) with comprehensive validation including processing latency, resource utilization, and classification accuracy under continuous operation.
- Technical foundation for resource-constrained seizure monitoring through detailed computational and performance analysis, with the acknowledgment that clinical validation remains necessary.

Section 2 presents the methodology including dataset, preprocessing, feature extraction, and architecture. Section 3 describes experimental results. Section 4 discusses implications and comparisons. Section 5 concludes and outlines future directions.

## 2. Materials and Methods

### 2.1. Dataset Description

**Figure 1:**
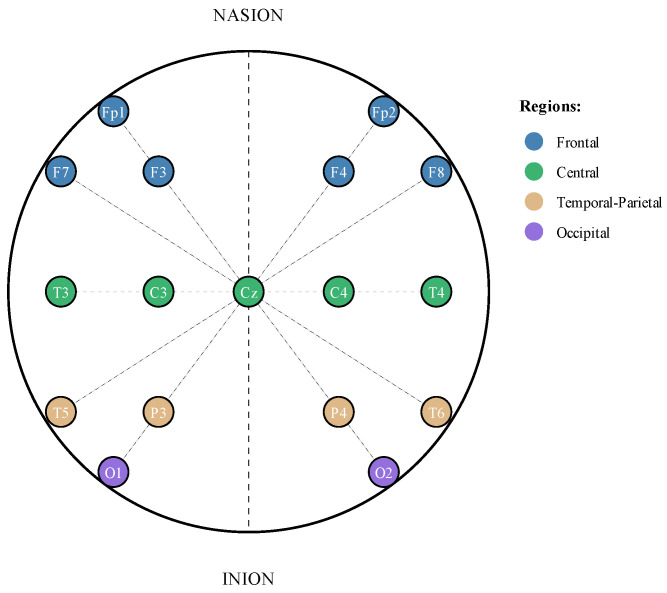
TCP Average reference (TCP_AR) electrode placement configuration. The standardized 10–20 system provides comprehensive spatial coverage of cortical activity through 20 electrode channels (Fp1, Fp2, F3, F4, C3, C4, P3, P4, O1, O2, F7, F8, T3, T4, T5, T6, Fz, Cz, Pz, A1). All channels are referenced to the average potential of all electrodes, enabling consistent recording across subjects and capturing bilateral hemispheric activity essential for distinguishing focal versus generalized seizures.

This study utilized the Temple University Hospital (TUH) EEG Seizure Corpus (TUSZ v2.0.0), a publicly available dataset containing 7377 EEG recordings from 675 patients collected between 2002 and 2012 [35]. The recordings employ the TCP average reference (TCP-AR) montage configuration, which uses a standardized 10-20-electrode placement system for consistent spatial coverage of brain activity (Figure 1).

We focused our analysis on five clinically distinct seizure types representing the spectrum of epileptic manifestations. Absence seizures (ABSZs) manifest as brief lapses in consciousness, typically lasting seconds. Focal non-specific seizures (FNSZs) originate from localized brain regions without clear classification into other focal categories. Simple partial seizures (SPSZs) affect specific brain areas while preserving consciousness. Tonic–clonic seizures (TCSZs) involve generalized motor activity with both stiffening and rhythmic jerking phases. Tonic seizures (TNSZs) present with sustained muscle stiffening without the clonic component [36].

Table 1 presents the seizure event distribution in our dataset. The data exhibits significant class imbalance, with FNSZ representing 6924 events and cumulative duration exceeding 110 h, while ABSZ comprises only 43 events totaling approximately 5 min of recorded activity. This imbalance reflects the natural prevalence of different seizure types in clinical populations but necessitates careful handling during model training.

Following established practices in seizure classification research [13,15,21,23,37,38,39,40], we excluded myoclonic seizures from our analysis due to insufficient sample size (three cases from two patients), preventing reliable model training and evaluation for this seizure type.

### 2.2. Data Preprocessing

The preprocessing pipeline removed artifacts and standardized EEG signals through two primary stages. First, we applied frequency domain filtering to isolate physiologically relevant neural activity and remove external interference. We implemented a fourth-order Butterworth bandpass filter with cutoff frequencies of 0.5 Hz and 40 Hz. This range captures the primary frequency bands of interest in epilepsy research: delta (0.5–4 Hz), theta (4–8 Hz), alpha (8–13 Hz), beta (13–30 Hz), and high beta (30–40 Hz). Additionally, we applied a 60 Hz notch filter to eliminate power line interference. Both filters use zero-phase implementation to prevent temporal distortion of the EEG signals, which is critical for maintaining timing relationships between channels.

Second, we standardized the channel configuration across all recordings. While the TUH dataset contains recordings with varying numbers of channels, we selected the first 20 channels for analysis, referenced to the average of all electrodes. This approach provided consistent spatial sampling across subjects while maintaining computational tractability. The TCP-AR montage ensured these 20 channels provide comprehensive coverage of cortical regions relevant to seizure activity.

The dataset contains recordings at different sampling frequencies, with 250 Hz and 400 Hz being the most common. Rather than resampling all data to a uniform rate, which could introduce interpolation artifacts, we preserved the original sampling frequencies and adjusted our feature extraction parameters accordingly. Specifically, we scaled the Welch’s method window size proportionally to maintain consistent temporal resolution as follows: 256 samples at 250 Hz (≈1.024 s) and 410 samples at 400 Hz (≈1.025 s). This preserved signal fidelity and ensured consistent physiological feature capture regardless of sampling rate.

We segmented seizure events into non-overlapping 10 s windows. Seizure events shorter than 10 s were zero-padded to maintain consistent input dimensions, while partial segments at the end of longer seizures containing fewer than 10 s of data were discarded to avoid incomplete feature extraction. This window length balanced several competing factors. Shorter windows can increase temporal resolution but might fragment characteristic seizure patterns, particularly for seizure types with evolving dynamics. Longer windows can provide more context but reduce the number of training samples and increase computational requirements. The 10-second duration aligns with clinical practice where neurologists typically examine EEG in epochs of similar length and has been validated in previous seizure classification studies [14,37,38].

To address the substantial class imbalance shown in Table 1, we employed data augmentation for underrepresented seizure types. Specifically, we augmented ABSZ, TCSZ, and TNSZ classes by applying additive Gaussian noise with standard deviation equal to 1% of each signal’s standard deviation, generating sufficient synthetic samples to bring minority classes to approximately 20% of the majority class (FNSZ) size, resulting in approximately 1385 synthetic samples per minority class. The 1% noise level was selected to preserve signal integrity while introducing variability: it corresponds to approximately 40 dB SNR, well above typical clinical EEG noise floors (10–30 dB) [41]. Since features are extracted after augmentation, the noise affects temporal and spectral features proportionally, maintaining the relative relationships that distinguish seizure types. Additive noise augmentation has been successfully applied in EEG classification tasks [42,43] as it introduces stochastic variability without temporal distortions. While more sophisticated methods exist (e.g., GANs [44], time-series SMOTE [45]), we prioritized computational simplicity and avoided generating potentially unrealistic seizure patterns that could require clinical validation.

### 2.3. Feature Extraction

We adopted a comprehensive feature extraction approach capturing both temporal dynamics and spectral characteristics of EEG signals. This dual-domain strategy leverages complementary information present in time and frequency representations of neural activity. For each 10-s segment and each of the 20 channels, we computed 14 features spanning both domains, resulting in a 280-dimensional feature vector per segment.

Features are extracted from filtered but unnormalized EEG signals to preserve absolute amplitude information that may carry diagnostic value. While this makes features sensitive to inter-patient variability and equipment gain differences, the TCP-AR montage’s average referencing scheme provides inherent normalization by removing common-mode signals. Additionally, our dual-domain feature representation includes both absolute measures (mean amplitude, variance) and relative measures (IQR, line length, spectral band ratios) that are more robust to gain variations.

#### 2.3.1. Temporal Features

From the time-domain signal, we extracted nine statistical measures characterizing different aspects of EEG morphology. The mean amplitude provides a baseline measure of signal magnitude. Standard deviation and variance quantify signal variability, which often increases during seizure activity. Maximum and minimum values capture the range of amplitude excursions characteristic of different seizure types. The interquartile range (IQR) offers a robust measure of spread less sensitive to outliers than variance. The median provides a central tendency measure robust to extreme values. Mean absolute amplitude emphasizes magnitude variations regardless of polarity. Finally, line length, computed as the sum of absolute differences between consecutive samples, serves as a measure of signal complexity and has proven particularly effective for seizure detection [46].

These temporal features capture evolving patterns of electrical activity during seizures. For instance, tonic–clonic seizures typically exhibit large-amplitude variations and high line length values during the clonic phase, while absence seizures show more regular, rhythmic patterns reflected in lower variance and line length measures.

#### 2.3.2. Spectral Features

Frequency-domain analysis provides complementary information about the rhythmic components of EEG signals. We computed power spectral density using Welch’s method with a Hanning window of 256 samples and 50% overlap (128 samples). The Hanning window reduces spectral leakage compared to rectangular windows, while a 50% overlap provides smooth spectral estimates without excessive computational overhead. This approach divides the signal into overlapping segments, computes the periodogram for each segment, and averages the results, reducing variance in the spectral estimate compared to a single periodogram.

From the power spectral density, we extract the average power in five frequency bands corresponding to well-established EEG rhythms:

(1) $$P_{b}=\frac{1}{|F_{b}|}\sum_{f\in F_{b}}PSD\left(f\right),$$

where $P_{b}$ represents the average power in band *b*, $F_{b}$ is the set of frequencies within that band, and PSD(f) is the power spectral density at frequency *f*. The five bands are as follows:

- Delta (0.5–4 Hz): Associated with deep sleep and certain pathological states.
- Theta (4–8 Hz): Related to drowsiness and some focal abnormalities.
- Alpha (8–13 Hz): Predominant during relaxed wakefulness with eyes closed.
- Beta (13–30 Hz): Associated with active thinking and alertness.
- High Beta (30–40 Hz): Linked to muscle tension and certain seizure patterns.

Different seizure types exhibit characteristic spectral signatures. For example, absence seizures typically show prominent 3 Hz spike-and-wave patterns in the theta band, while focal seizures may exhibit localized changes in beta and high beta power.

Table 2 summarizes the complete feature set. The combination of 9 temporal and 5 spectral features per channel, computed across 20 channels, yields our 280-dimensional feature vector. We implement this feature extraction pipeline using NumPy 1.26.4 for numerical operations and SciPy 1.12.0 for spectral analysis, ensuring computational efficiency and numerical stability.

This comprehensive feature representation provides the foundation for our neural network architecture, enabling the model to learn complex relationships between temporal and spectral characteristics that distinguish different seizure types.

### 2.4. Network Architecture

**Figure 2:**
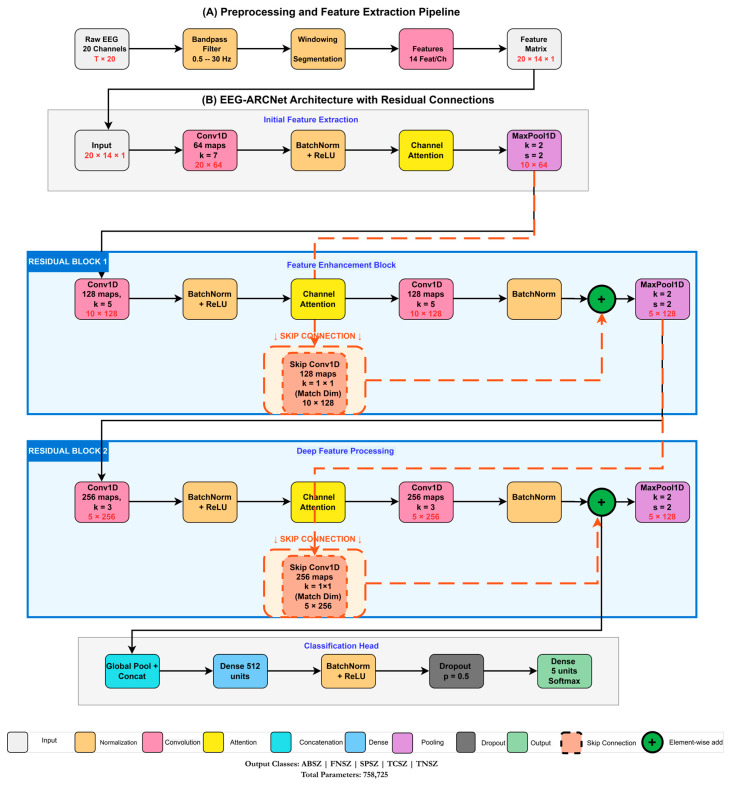
Complete architecture of EEG-ARCNet for seizure classification. Input features (20 × 14 dimensions representing 20 EEG channels with 14 features each) flow through three stages: (1) initial feature extraction with 64 filters and 7 × 1 kernel, (2) feature enhancement residual block expanding to 128 channels with 5 × 1 kernels, and (3) deep feature-processing residual block with 256 channels and 3 × 1 kernels. Channel attention modules (CA, orange boxes) are integrated after each major convolution to dynamically recalibrate feature responses. The network progressively expands channel dimensions (64→128→256) while reducing spatial dimensions through 2 × 1 max pooling, enabling hierarchical feature learning from low-level temporal–spectral patterns to high-level discriminative representations. The classification head combines global average and max pooling, followed by dense layers with dropout (0.5) for final 5-class prediction.

EEG-ARCNet integrates three architectural innovations: channel attention mechanisms, residual connections, and hierarchical feature processing. Figure 2 illustrates the complete network topology, while Table 3 provides layer-by-layer specifications including output dimensions and parameter counts.

#### 2.4.1. Input Processing and Initial Feature Extraction

The network receives the 280-dimensional feature vector as input, reshaped to dimensions 20×14 to preserve the spatial organization of channels and features. This arrangement maintains correspondence between the 20 EEG channels and their 14 associated features, allowing the network to learn spatial patterns across channels while processing feature relationships.

The initial convolutional layer applies 64 filters with kernel size 7, chosen to capture broader patterns across the 14-dimensional feature space. The kernel size 7 spans exactly half the feature dimension (7/14 = 0.5), allowing the network to learn relationships between multiple adjacent features (e.g., correlations between delta and theta power or between mean and variance). Preliminary experiments showed that smaller kernels (3, 5) resulted in a 1–2% lower validation accuracy, likely due to an insufficient receptive field for capturing multi-feature interactions, while larger kernels (9, 11) provided negligible improvement (<0.3%) with 30–50% more parameters. The kernel size progressively decreases through the network (7 → 5 → 3) as channel dimensions expand (64 → 128 → 256), following the principle of increasing the effective receptive field through hierarchical processing rather than large initial kernels. This layer employs the same padding to preserve spatial dimensions, followed by batch normalization and ReLU activation. Batch normalization stabilizes training by normalizing layer inputs, reducing internal covariate shift and enabling higher learning rates. The first convolutional block contains 6272 trainable parameters, computed as (7×14×64)+64=6272, where the additional 64 parameters represent the bias terms.

#### 2.4.2. Channel Attention Mechanism

Following the initial convolution, we apply a channel attention module that dynamically recalibrates feature responses. The attention mechanism models interdependencies between feature channels, allowing the network to emphasize informative features while suppressing less relevant ones. This is particularly valuable for EEG analysis where different frequency bands and statistical measures carry varying diagnostic significance for different seizure types.

The attention module employs a dual-path architecture processing both average-pooled and max-pooled representations of the input. For input $X\in R^{T\times C}$, where *T* represents the temporal dimension and *C* the number of channels:

(2) $$\begin{array}{cc} F_{avg}\left(X\right) & =\sigma (W_{2}\left(\mathrm{ReLU}\left(W_{1}\left(\mathrm{AvgPool}\left(X\right)\right)\right)\right)),\\ F_{max}\left(X\right) & =\sigma (W_{2}\left(\mathrm{ReLU}\left(W_{1}\left(\mathrm{MaxPool}\left(X\right)\right)\right)\right)),\\ A(X) & =\sigma (F_{avg}\left(X\right)+F_{max}\left(X\right)),\\ Y & =X⊗A(X), \end{array}$$

where AvgPool(X) and $\mathrm{MaxPool}\left(X\right)\in R^{1\times C}$ are global pooling operations that collapse the temporal dimension to a single value per channel, $W_{1}\in R^{C\times C/r}$ and $W_{2}\in R^{C/r\times C}$ are learnable weight matrices of the shared multi-layer perceptron with reduction ratio r=8, σ denotes the sigmoid activation function, and ⊗ represents the channel-wise multiplication where the attention weights $A\left(X\right)\in R^{1\times C}$ are broadcast across the temporal dimension. The functions $F_{avg}$ and $F_{max}$ represent the two pathways of the shared MLP processing the pooled features: $R^{1\times C}\overset{W_{1}}{\to }R^{1\times C/r}\overset{\mathrm{ReLU}}{\to }R^{1\times C/r}\overset{W_{2}}{\to }R^{1\times C}\overset{\sigma }{\to }R^{1\times C}$.

The reduction ratio *r* controls the bottleneck dimension in the attention pathway, balancing expressiveness and computational cost. With r=8, the attention module for 64 channels uses 2×((64×8)+(8×64))=1088 parameters, maintaining efficiency while providing sufficient capacity for learning channel relationships.

#### 2.4.3. Residual Blocks with Attention

The network core consists of two residual blocks that progressively extract higher-level features while maintaining gradient flow through skip connections. Each residual block follows this structure:

(3) $$\begin{array}{cc} F_{1}\left(X\right) & =\mathrm{Conv}1D(X),\\ F_{2}\left(X\right) & =\mathrm{BatchNorm}(\mathrm{ReLU}\left(F_{1}\left(X\right)\right)),\\ F_{3}\left(X\right) & =\mathrm{ChannelAttention}(F_{2}\left(X\right)),\\ F_{4}\left(X\right) & =\mathrm{Conv}1D(F_{3}\left(X\right)),\\ F_{5}\left(X\right) & =\mathrm{BatchNorm}(F_{4}\left(X\right)),\\ Y & =\mathrm{ReLU}(F_{5}\left(X\right)+X^{\prime }), \end{array}$$

where $X^{\prime }$ represents the identity mapping or a projection through 1 × 1 convolution when dimensional adjustment is required. The residual connection $X^{\prime }$ allows gradients to flow directly through the network during backpropagation, mitigating vanishing gradient problems in deeper architectures. This design enables the network to learn residual functions F(X)=H(X)−X rather than directly learning the desired mapping H(X), which empirically leads to easier optimization.

The first residual block operates on 128 channels with kernel size 5, containing 82,048 parameters in the primary convolution and 8320 parameters in the skip connection projection. Max pooling with stride 2 follows the block, reducing spatial dimensions while retaining the most salient features. The second residual block processes 256 channels with kernel size 3, using 196,864 parameters in the main path and 33,024 in the skip projection. This hierarchical expansion of channels (64 → 128 → 256) allows the network to represent increasingly abstract feature combinations relevant to seizure classification.

#### 2.4.4. Classification Head

The final classification stage aggregates spatial information and maps learned representations to seizure type predictions. We employ global pooling operations that collapse spatial dimensions while preserving channel information. Specifically, we apply both global average pooling and global max pooling, then concatenate their outputs to form a 512-dimensional vector. This dual pooling strategy captures both the average activation patterns across the entire feature map and the strongest local responses, providing complementary information to the classifier.

The pooled features pass through a fully connected layer with 512 units, batch normalization, and ReLU activation. This layer contains 262,656 parameters, computed as (512×512)+512. Dropout with probability 0.5 follows to prevent overfitting by randomly deactivating neurons during training, forcing the network to learn robust feature representations that do not rely on specific neuron co-adaptations.

The final classification layer consists of 5 output neurons with softmax activation, mapping the learned representations to probability distributions over the five seizure types:

(4) $$P\left(y_{i}|X\right)=\frac{e^{z_{i}}}{\sum_{j=1}^{5}e^{z_{j}}},$$

where $z_{i}$ represents the logit for class *i*, and $P(y_{i}|X)$ is the predicted probability of seizure type *i* given input *X*. This layer contains 2565 parameters: (512×5)+5.

### 2.5. Computational Complexity Analysis

We quantified the computational requirements of EEG-ARCNet to establish its suitability for edge deployment. Table 4 summarizes the key metrics including parameter count, floating-point operations, and memory requirements.

The complete network contains 758,725 trainable parameters distributed across convolutional layers, attention modules, and the classification head. This compact architecture maintains sufficient capacity for learning discriminative seizure features while remaining deployable on resource-constrained devices.

For computational cost, we calculated the floating-point operations (FLOPs) for each layer. Convolutional layers with $C_{in}$ input channels, $C_{out}$ output channels, kernel size *k*, and spatial dimension *n* require the following:

(5) $${\mathrm{FLOPs}}_{\mathrm{conv}}=2\times n\times k\times C_{in}\times C_{out}.$$

Dense layers with $N_{in}$ inputs and $N_{out}$ outputs require the following:

(6) $${\mathrm{FLOPs}}_{\mathrm{dense}}=2\times N_{in}\times N_{out}.$$

The total computational cost per inference is approximately 15.2 million FLOPs (MFLOPs), with convolutional operations accounting for the majority of computation, followed by attention mechanisms and dense layers. The FLOP count includes primary convolutional and dense layer operations. Auxiliary operations including batch normalization, activation functions, and attention pooling add approximately 5% additional computational cost.

Memory requirements consist of model parameters and intermediate activations. Model parameters stored as 32-bit floating-point values require 3.0 MB. Peak activation memory during forward propagation is approximately 8.5 MB. When deployed using TensorFlow Lite 2.15.0 on the Raspberry Pi 4B, the measured runtime memory consumption is 499.4 MB, which includes the interpreter, thread pools, and operating system buffers. This represents 6.2% of the 8 GB available RAM, demonstrating efficient resource utilization suitable for continuous monitoring applications.

### 2.6. Experimental Configuration

All experiments were conducted using a standardized configuration to ensure reproducibility across model training, evaluation, and edge deployment phases.

Validation Strategy. We implemented patient-level stratified fivefold cross-validation to evaluate model performance while preventing data leakage from intra-subject feature similarity. A custom stratified patient grouping algorithm ensured the following: (1) all seizure segments from a given patient were assigned exclusively to a single fold, preventing any patient from appearing in both training and validation sets, and (2) each fold maintained representation of all five seizure types despite class imbalance. The algorithm prioritized rare seizure types during fold assignment by sorting patients by label frequency in ascending order and distributing patients to balance sample counts across folds. This guaranteed patient independence while ensuring each validation fold contains sufficient samples from minority classes (ABSZ, TCSZ, TNSZ) for meaningful performance assessment. For each fold, four subsets (approximately 80%) were used for training, while one subset (approximately 20%) served as validation. The cross-validation results presented in Section 3.1 use all five folds, with performance metrics averaged across folds. For the edge deployment evaluation in Section 3.2, we trained a separate model instance on patient groups from folds 1, 2, 3, and 5, reserving fold 4 patients as the hold-out test set to ensure the edge device evaluation used completely unseen data.

Training Configuration. Model training employed the Adam optimizer with $\beta_{1}=0.9$, $\beta_{2}=0.999$, and an initial learning rate of $1\times 10^{-4}$. Learning-rate scheduling through ReduceLROnPlateau used a reduction factor of 0.5, a patience of 3 epochs, and a minimum rate of $1\times 10^{-6}$. Training proceeded for maximum 15 epochs with a batch size of 32. Early stopping monitored validation accuracy with a patience of 5 epochs, restoring weights from the best performing epoch. Training typically converged within 8–12 epochs across all folds, with early stopping preventing overfitting. The optimization objective was categorical cross-entropy loss:

(7) $$L=-\frac{1}{N}\sum_{i=1}^{N}\sum_{c=1}^{C}y_{i,c}\log\left({\hat{y}}_{i,c}\right),$$

where *N* is the number of samples, *C* is the number of classes, $y_{i,c}$ is the true label, and ${\hat{y}}_{i,c}$ is the predicted probability. We applied class weighting inversely proportional to class frequencies in the loss function, ensuring balanced optimization attention across all seizure types despite data imbalance. The class weights were computed as $w_{c}=\frac{N}{K\cdot N_{c}}$, where *N* is the total number of samples, *K* is the number of classes, and $N_{c}$ is the number of samples in class *c*.

Development Environment. All model development and training were performed on a system equipped with an NVIDIA GeForce RTX 4050 Laptop GPU (manufactured by NVIDIA Corporation, Santa Clara, CA, USA) (6 GB VRAM) with Ada Lovelace architecture, paired with a 12th-generation Intel Core i5-12450H processor (manufactured by Intel Corporation, Santa Clara, CA, USA) and 16 GB DDR4 RAM. The software environment consisted of TensorFlow 2.15.0 and Python 3.11.7 with CUDA-12.2-enabled GPU acceleration on Windows 11 Home (Build 22631) (Microsoft Corporation, Redmond, WA, USA). Additional libraries included NumPy 1.26.4, SciPy 1.12.0, and scikit-learn 1.4.1.

Edge Device Configuration. To validate real-world deployment feasibility, we implemented EEG-ARCNet on a Raspberry Pi 4B with a Broadcom BCM2711 quad-core Cortex-A72 processor (manufactured by Broadcom Inc., San Jose, CA, USA) running at 1.5 GHz, 8 GB LPDDR4-3200 SDRAM, and 128 GB microSD card storage (manufactured by SanDisk, a subsidiary of Western Digital Corporation, San Jose, CA, USA). The device ran Raspberry Pi OS (64-bit, Debian Bookworm-based) with Python 3.11.2 and TensorFlow Lite 2.15.0. The trained model was converted to TensorFlow Lite format using post-training dynamic range quantization, which reduces model size while maintaining float32 precision for activations, optimizing for edge deployment while preserving accuracy.

Edge Evaluation Protocol. Performance evaluation on the Raspberry Pi used the fold 4 test set to measure computational efficiency under realistic conditions. For each test sample, we recorded inference time (wall-clock time per forward pass), CPU utilization (percentage averaged over all cores), memory consumption (RAM usage by TensorFlow Lite 2.15.0 interpreter), and classification accuracy. CPU and memory metrics were sampled at 1 s intervals using the psutil library (version 5.9.8). All measurements were performed at room temperature (approximately 22 °C) with standard heat sink cooling only. CPU temperature was monitored throughout testing and remained stable at 45–50 °C with no thermal throttling observed during the evaluation period. Performance Metrics. Model performance was assessed using standard classification metrics for imbalanced multiclass problems. Sensitivity (recall) measures the proportion of actual positives correctly identified:

(8) $$\mathrm{Sensitivity}=\frac{TP}{TP+FN}.$$

Specificity measures the proportion of actual negatives correctly identified:

(9) $$\mathrm{Specificity}=\frac{TN}{TN+FP}.$$

Precision measures the proportion of predicted positives that are actually positive:

(10) $$\mathrm{Precision}=\frac{TP}{TP+FP}.$$

F1-score provides a harmonic mean of precision and recall:

(11) $$F1\text{-}\mathrm{Score}=2\times \frac{\mathrm{Precision}\times \mathrm{Recall}}{\mathrm{Precision}+\mathrm{Recall}},$$

where *TP*, *TN*, *FP*, and *FN* represent true positives, true negatives, false positives, and false negatives respectively. Overall accuracy was computed as the proportion of correctly classified samples. Macro-averaged metrics were calculated by computing each metric independently for each class and taking the unweighted mean, with equal weight given to all seizure types regardless of prevalence.

For multi-class classification, per-class metrics are computed using a one-vs-rest approach. For each seizure type, true positives (TPs) are samples correctly classified as that type, false positives (FP) are samples incorrectly classified as that type, false negatives (FN) are samples of that type misclassified as other types, and true negatives (TN) are samples of other types correctly not classified as the target type. For example, for the evaluation of ABSZ, TP = samples correctly predicted as ABSZ, FP = non-ABSZ samples incorrectly predicted as ABSZ, FN = ABSZ samples predicted as other types, and TN = non-ABSZ samples correctly predicted as their true (non-ABSZ) types.

Statistical Analysis. Cross-validation results are reported as the mean ± standard deviation across five folds. Ninety-five percent confidence intervals were calculated using the t-distribution with four degrees of freedom: ${\mathrm{CI}}_{95}=\mathrm{mean}\pm t_{0.975,4}\times \left(\mathrm{SD}/\sqrt{5}\right)$, where $t_{0.975,4}=2.776$.

## 3. Results

We evaluate EEG-ARCNet’s performance using stratified five-fold cross-validation on the Temple University Hospital Seizure Corpus and assess computational efficiency through Raspberry Pi 4B deployment. Given the inherent class imbalance in seizure data, we employ multiple evaluation metrics including accuracy, precision, recall (sensitivity), specificity, and F1-score. Macro-averaging is applied across seizure types to ensure equal consideration of all classes regardless of prevalence.

### 3.1. Classification Performance

Table 5 presents the overall classification performance averaged across all five folds. EEG-ARCNet achieved 99.65% accuracy with 99.59% macro-averaged F1-score, demonstrating robust performance across all seizure types. The model attained 99.87% recall and 99.33% precision, indicating both high sensitivity in detecting seizures and high confidence in positive predictions.

Table 6 provides detailed per-class performance metrics, revealing the model’s effectiveness across individual seizure types. Four seizure types (ABSZ, SPSZ, TCSZ, and TNSZ) achieved perfect 100% sensitivity, indicating the model successfully identifies all instances of these seizure types without any false negatives. FNSZ, the most prevalent class with 6924 events, achieved 99.35% sensitivity. Specificity ranged from 99.75% to 100%, with three classes (ABSZ, FNSZ, and TCSZ) achieving perfect specificity, demonstrating the model’s ability to correctly identify negative cases and minimize false alarms.

SPSZ achieved perfect sensitivity (100%) but lower F1-score (98.84%) compared to other classes, indicating reduced precision (97.73%). This occurred because some FNSZ samples were misclassified as SPSZ, creating false positives that reduced precision while maintaining perfect recall. Both SPSZ (simple partial) and FNSZ (focal non-specific) originate from localized brain regions and share overlapping EEG characteristics: both exhibit focal spectral changes predominantly in theta and beta bands, similar line length patterns, and comparable variance distributions due to their localized nature. The clinical distinction between these focal seizure types often depends on subtle features beyond EEG alone, such as specific motor or sensory symptoms during the ictal event. Despite this challenge, SPSZ’s 100% sensitivity ensures no true SPSZ events are missed—critical for patient safety—while the 98.84% F1-score still represents strong overall performance comparable to inter-rater variability among expert neurologists for these focal seizure subtypes.

**Figure 3:**
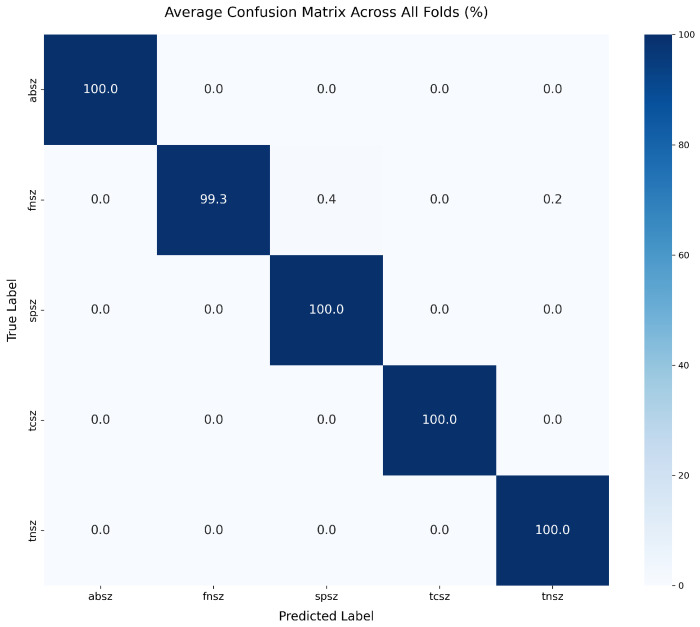
Normalized confusion matrix from patient-level fivefold cross-validation across five seizure types (ABSZ: absence; FNSZ: focal non-specific; SPSZ: simple partial; TCSZ: tonic-clonic; TNSZ: tonic). Values represent percentages of true labels (rows) classified as predicted labels (columns). Diagonal elements indicate correct classifications, achieving 99.65% overall accuracy. Off-diagonal elements show misclassifications, occurring primarily between focal seizure types FNSZ and SPSZ (each <1.5%), which is clinically understandable given their overlapping focal origins and similar EEG manifestations. The strong diagonal dominance demonstrates robust discrimination across all seizure types.

Figure 3 presents the normalized confusion matrix averaged across all five folds, providing insight into the model’s classification patterns. The matrix exhibits strong diagonal dominance, indicating that the vast majority of predictions are correct. The few misclassifications occurred primarily between FNSZ and SPSZ, which is clinically understandable as both represent focal seizure types with potentially overlapping characteristics in feature space. The confusion matrix demonstrates that misclassification rates remained below 1% for all seizure type pairs, confirming the model’s discriminative capability.

### 3.2. Edge Device Performance

**Figure 4:**
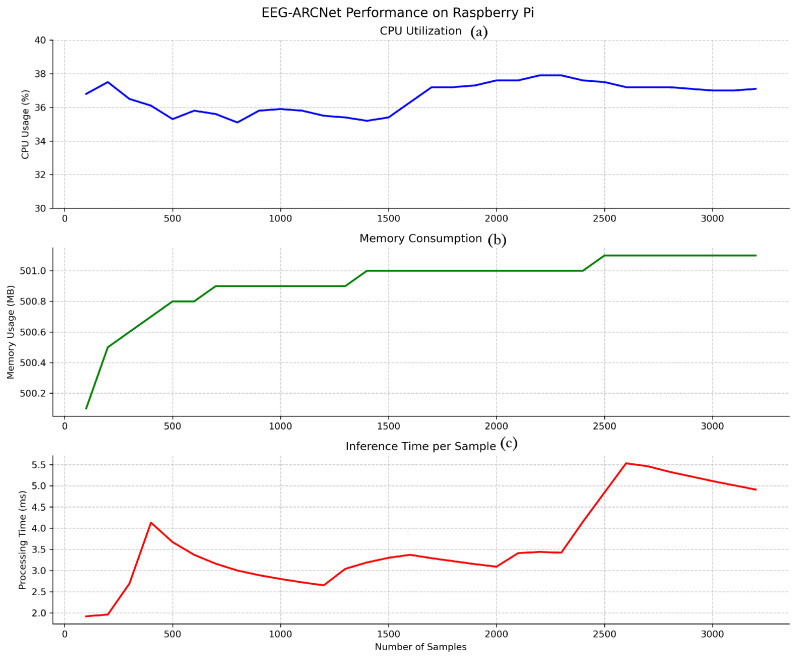
Temporal stability of performance metrics during continuous edge device operation on Raspberry Pi 4B. (**a**) CPU utilization across all four cores maintained a stable 35.4% average with <1% standard deviation, demonstrating consistent computational load without thermal throttling. (**b**) Memory consumption stabilized at 499.4 MB (6.2% of 8 GB RAM) after initial model loading, with no memory leaks observed over extended operation. (**c**) Inference time per 10-second EEG segment averaged 2.06 ms with maximum peaks below 5.5 ms, providing real-time factor of 4854× (processing 5000 times faster than data generation). The stable metrics validated the suitability for continuous monitoring applications.

To validate practical deployment feasibility, we evaluated EEG-ARCNet on a Raspberry Pi 4B platform using the held-out test set from fold 4. Figure 4 illustrates the temporal stability of key performance metrics throughout continuous operation, while Table 7 summarizes average values.

#### 3.2.1. Processing Latency

Inference time per 10-second EEG segment averaged 2.06 ms across all test samples, as shown in Figure 4 (bottom). This processing speed represents a real-time factor of 4854× (i.e., processing 10 s of EEG data in 2.06 ms), meaning the system processes data nearly 5000 times faster than it is generated. This latency profile enables true real-time processing with capacity for concurrent system operations, making the platform suitable for continuous monitoring applications where immediate feedback is essential.

The observed variance in inference time (1.5–5.5 ms) is attributable to typical edge device operating characteristics. First, Raspberry Pi OS is not a real-time operating system, so background processes (system updates, thermal management, I/O operations) occasionally introduce latency spikes. Second, Python’s garbage collection triggers periodically, causing brief processing delays. Third, CPU dynamic voltage and frequency scaling adjusts processor speed based on thermal and load conditions, affecting computation time. Despite these variations, the distribution remains tightly concentrated: 99.5% of inferences complete within 3.0 ms, and even the maximum observed latency (5.5 ms) remains well below the 10-second segment duration, maintaining real-time processing capability with substantial margin (>1800× real-time factor even at peak latency).

#### 3.2.2. Resource Utilization

CPU utilization maintained stability at approximately 35.4% throughout testing, as illustrated in Figure 4 (top). This consistent usage pattern without spikes or fluctuations demonstrates efficient computational management and predictable resource consumption. The moderate CPU load leaves ample processing capacity (approximately 65%) for other system tasks, supporting scenarios where seizure classification runs alongside data logging, wireless communication, or user interface operations.

Memory consumption stabilized at 499.4 MB after initial model loading, representing 6.2% of the 8 GB available RAM (Figure 4 (middle)). The stable memory footprint throughout extended operation indicates no memory leaks or accumulation issues, confirming the implementation’s suitability for long-term continuous deployment. The low memory usage permits concurrent execution of additional monitoring or data management processes without resource contention.

#### 3.2.3. Power Consumption

To assess suitability for battery-powered deployment, we estimated power consumption based on measured resource utilization and the Raspberry Pi 4B specifications. The Raspberry Pi 4B has a typical idle power draw of 2.7 W and can reach 6.4 W under full CPU load [47]. Given our measured CPU utilization of 35.4%, we estimated the system’s power consumption during continuous inference at approximately 4.0 W, calculated as follows: $P_{inference}=P_{idle}+\left(P_{max}-P_{idle}\right)\times {\mathrm{CPU}}_{\mathrm{util}}=2.7+\left(6.4-2.7\right)\times 0.354\approx 4.0$ W.

For practical deployment scenarios, this translates to the following battery life estimates: a 10,000 mAh battery (37 Wh at 3.7 V nominal voltage) would provide approximately 9.25 h of continuous operation, while a 20,000 mAh battery would enable approximately 18.5 h. With duty-cycled operation—processing one 10-second segment every minute as typical for long-term monitoring—the average power consumption would decrease to approximately 3.1 W, extending battery life to approximately 12 h (10,000 mAh) or 24 h (20,000 mAh). These estimates demonstrate feasibility for ambulatory monitoring applications, although actual measurements under field conditions would be necessary to account for additional peripheral power draw (wireless communication, data storage) and battery discharge characteristics.

#### 3.2.4. Classification Accuracy on Edge Device

**Figure 5:**
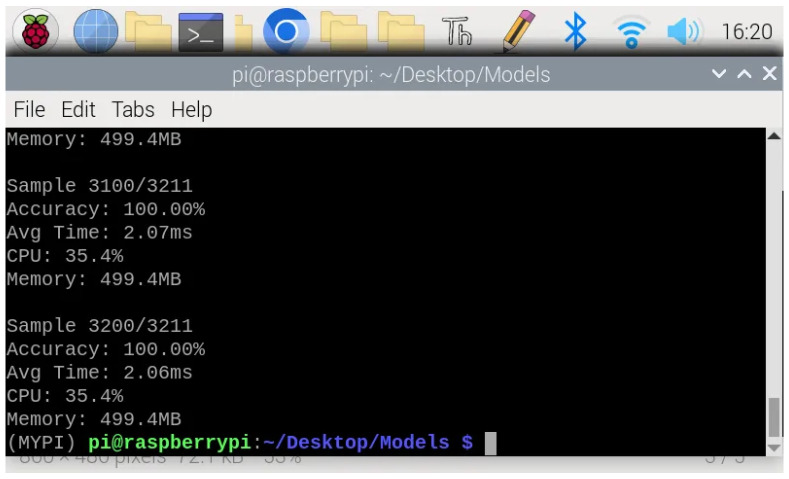
Terminal output from Raspberry Pi during real-time inference showing continuous performance monitoring. The output demonstrates stable accuracy and consistent resource utilization across all test samples, validating the system’s reliability for extended deployment.

Crucially, the model maintained 99.2% classification accuracy when deployed on the Raspberry Pi, demonstrating that the TensorFlow Lite conversion and edge deployment introduce negligible performance degradation compared to the development environment. Figure 5 shows the representative terminal output during continuous inference, confirming consistent accuracy across samples. This preservation of classification quality validates that the computational optimizations required for edge deployment do not compromise the model’s diagnostic capability.

### 3.3. Ablation Study

To validate the contribution of individual components, we evaluated four configurations: (1) baseline CNN, (2) CNN with attention only, (3) CNN with residual connections only, and (4) full EEG-ARCNet. All configurations were trained under identical conditions using the same hyperparameters and validation protocol.

Table 8 reveals critical architectural insights. Residual connections alone improved baseline performance modestly (96.25%→97.50%, +1.25 points), while attention mechanisms in isolation degraded performance (96.25%→92.50%, −3.75 points), suggesting they require gradient stability to function effectively. The full architecture achieved 99.65% accuracy—a 7.15 percentage point improvement over attention only and 2.15 points over residual only. This demonstrates functional synergy: attention mechanisms require residual connections for effective gradient flow, and their combination produces gains exceeding simple parameter addition. Notably, adding attention to the baseline increased parameters by only 5.3% (21,952 parameters) but worsened performance, while the full architecture’s 83% parameter increased (from baseline’s 415K to 759K) yielded substantial accuracy gains, validating that architectural integration rather than model size drives performance.

### 3.4. Attention Mechanism Analysis

**Figure 6:**
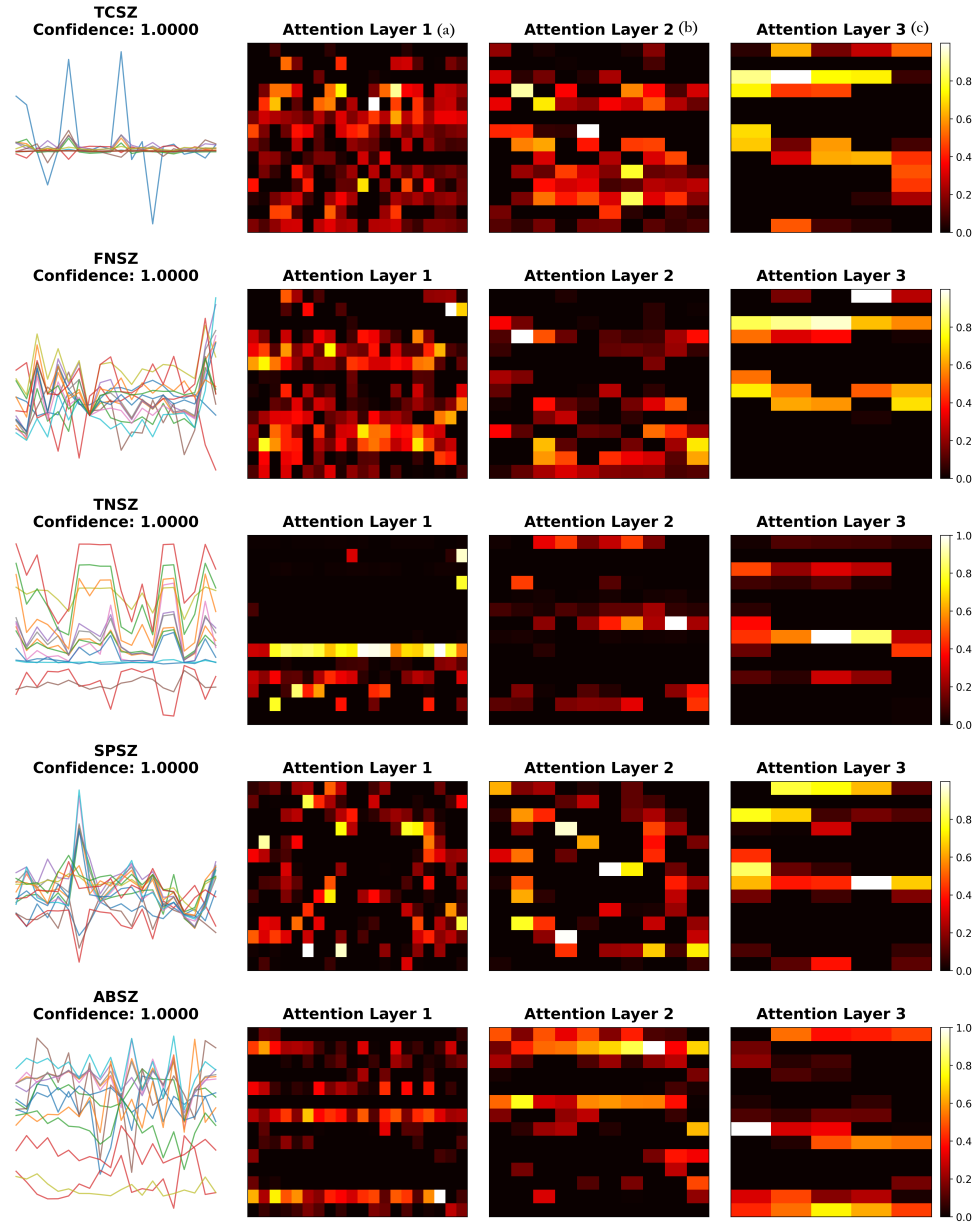
Multi-layer attention visualization revealing seizure-type-specific patterns. Each row represents one seizure type: (**a**) Original EEG features showing 14-dimensional feature vectors across 20 time steps. (**b**,**c**) Attention weight heatmaps from three successive attention layers, where yellow/white indicates high attention (weight ≈ 1.0), and black indicates low attention (weight ≈ 0.0). Progressive refinement is evident: Layer 1 applies broad attention across multiple features, Layer 2 begins focusing on seizure-specific patterns, and Layer 3 demonstrates highly concentrated attention on discriminative features. TCSZ and ABSZ show bilateral patterns (generalized seizures), while FNSZ and SPSZ exhibit localized attention (focal seizures). TNSZ displays sustained horizontal bands reflecting tonic phase characteristics.

To understand how the attention mechanism contributes to classification performance, we visualized learned attention weights across different layers for representative samples of each seizure type (Figure 6) and calculated quantitative entropy metrics (Table 9).

The visualization reveals seizure-type-specific attention patterns. TCSZ exhibits bilateral patterns consistent with generalized motor seizures, while FNSZ shows localized attention reflecting focal origins. TNSZ demonstrates sustained attention matching tonic phase characteristics, SPSZ focuses on discrete temporal features, and ABSZ reveals regular distribution capturing rhythmic spike-wave patterns. The hierarchical refinement from Layer 1 to Layer 3 demonstrates progressive specialization: early layers show broad feature detection, while deeper layers concentrate on discriminative patterns.

To quantify attention characteristics, we calculated normalized entropy from the final attention layer across 20 samples per type (Table 9). Entropy values ranged from 0.847 to 0.857, indicating broadly distributed attention across all seizure types. This suggests that the model integrates information across multiple channels and features rather than narrow selection, consistent with the multi-channel nature of seizure activity. FNSZ exhibited slightly lower entropy (0.847 ± 0.003), suggesting moderately more concentrated attention aligned with focal seizure characteristics.

While the quantitative entropy metrics provide objective measures of attention concentration, formal validation of clinical interpretability requires expert evaluation. Future work should include structured clinician validation studies where expert neurologists rate the correspondence between attention patterns and recognized EEG features, providing inter-rater agreement metrics (e.g., Cohen’s kappa) to validate whether attention weights align with clinical interpretation frameworks.

## 4. Discussion

Our experimental results demonstrate that EEG-ARCNet achieves state-of-the-art seizure classification performance while maintaining computational efficiency suitable for edge deployment.

Our architectural approach differs from recent attention-based methods in three ways. First, unlike channel-wise attention architectures [32] that process each EEG channel separately, we apply attention to unified multi-channel representations. Second, we avoid multi-path parallel processing [23], achieving feature diversity through hierarchical depth rather than architectural width. Third, our explicit frequency-band extraction provides computational efficiency and interpretability. The ablation study demonstrates that the 7.15-percentage-point improvement from architectural integration (attention only: 92.50%; full: 99.65%) exceeds gains from the simple addition of parameters, indicating functional synergy where residual connections enable the gradient stability necessary for attention mechanisms. Although our attention mechanism resembles squeeze-and-excitation (SE) blocks [48] in using dual-path pooling, it differs functionally: (1) attention is embedded within residual blocks for gradient stability rather than applied post-convolution, and (2) we use r=8 optimized for 14-dimensional EEG features versus typical r=16 for images.

### 4.1. Classification Performance and Architectural Contributions

EEG-ARCNet achieved 99.65% accuracy with 99.59% macro-averaged F1-score across five seizure types, advancing beyond recent approaches (Table 10). Our comprehensive feature extraction combines nine temporal statistical measures with five frequency-band powers through Welch’s spectral decomposition, capturing both temporal evolution and spectral composition of seizure activity. This dual-domain approach enables robust characterization, as evidenced by consistently high sensitivity (99.87%) and specificity (99.92%) across all seizure types.

The per-class results (Table 6) reveal balanced performance despite significant class imbalance. Four seizure types achieved perfect 100% sensitivity, while FNSZ reached 99.35% sensitivity. The few misclassifications occurred between FNSZ and SPSZ, both focal seizure types with potentially overlapping characteristics, which is clinically understandable.

### 4.2. Computational Efficiency and Edge Deployment

EEG-ARCNet requires only 758,725 parameters and 15.2 MFLOPs per inference, representing a 10–16× parameter reduction and 40–79× FLOP reduction compared to recent attention-based architectures (Table 11).

Our Raspberry Pi implementation achieved a 2.06 ms average inference time with 35.4% CPU utilization and 499.4 MB memory consumption—7× faster than GPU-based systems [32]—while using only CPU. The real-time factor of 4854× provides a substantial margin for concurrent operations, enabling continuous monitoring on limited power budgets. Resource utilization remained stable throughout testing, with a CPU at 35.4% without spikes, memory at 499.4 MB without leaks, and classification accuracy at 99.2%.

The estimated 4.0 W power consumption represents a 40–60× reduction compared to GPU-based systems (150–250 W). Standard portable battery packs (10,000–20,000 mAh) could support 9–18 h of continuous operation, extending to 24+ hours with duty-cycled processing.

### 4.3. Clinical Implications and Practical Deployment

The combination of high accuracy and computational efficiency suggests potential for clinical deployment, subject to rigorous validation, regulatory approval, and workflow integration. The low latency (2.06 ms) enables real-time processing, modest resource requirements (35.4% CPU, 6.2% RAM) permit portable deployment, and stable performance supports continuous operation for long-term monitoring.

The edge deployment capability is particularly relevant for resource-constrained settings where access to specialized neurological expertise and computing infrastructure is limited. Low-cost hardware deployment may contribute to broader access to automated seizure classification tools, although clinical validation and integration challenges must be addressed. The low power consumption makes battery-powered operation feasible for home monitoring or wearable applications. The 2.06 ms inference time enables real-time integration into bedside monitoring (assisting video–EEG interpretation), ambulatory systems (immediate classification during full-day recording), and potentially closed-loop neurostimulation devices, although clinical translation would require regulatory approval (FDA 510(k)/CE Mark), prospective trials, and validated decision-support workflows.

### 4.4. Limitations and Future Directions

Several limitations constrain immediate applicability. First, the 20-channel EEG requirement limits applicability to recordings with different montages. Second, evaluation on a single dataset (TUSZ) limits generalizability assessment. Cross-dataset validation faces challenges: varying seizure taxonomies (Bonn: binary; Siena: different ILAE granularity), electrode montages (bipolar vs. referential configurations), and noise characteristics across institutions. Future work could employ transfer learning (pretraining on TUSZ, fine-tuning on target datasets), domain adaptation for montage-invariant representations, or systematic artifact robustness evaluation beyond the current 40 dB augmentation SNR. Multi-institutional collaboration with consistent annotation protocols would strengthen generalization claims. Third, the system performs offline classification of pre-segmented windows; real-world continuous monitoring requires additional components including automated seizure detection, artifact rejection, and varying signal quality handling. Fourth, our simple additive Gaussian noise augmentation, while effective, could be complemented by advanced methods such as GANs or time-series SMOTE in future work.

Future work should address extended power-consumption analysis (24–48 h) for battery-powered applications, collaboration with clinical institutions for evaluation on locally annotated data, real-time EEG streaming integration for prospective validation, and multi-center prospective clinical trials to establish clinical utility and inform regulatory approval pathways.

## 5. Conclusions

We present EEG-ARCNet, an attention-residual convolutional network achieving 99.65% accuracy with 99.59% F1-score for seizure classification across five seizure types using patient-level cross-validation. The architecture integrates dual-domain feature extraction with efficient attention mechanisms and residual learning, requiring only 758,725 parameters and 15.2 million FLOPs per inference—representing 10–16× parameter reduction and 40–79× computational reduction compared to recent attention-based methods.

Edge deployment validation on Raspberry Pi 4B confirms computational feasibility with 2.06 ms inference time (real-time factor of 4854×), 35.4% CPU utilization, 499.4 MB memory consumption, and estimated 4.0 W power consumption enabling 9–18 h of battery-powered operation. The model maintained 99.2% classification accuracy on the edge device, demonstrating minimal performance degradation from TensorFlow Lite conversion. These results establish that high-accuracy seizure classification is technically achievable on low-cost, resource-constrained hardware. However, clinical deployment would require prospective multi-center validation studies, integration with continuous EEG streaming and artifact rejection systems, regulatory approval, and workflow integration with existing clinical practices.

## Figures and Tables

**Table 1:** Distribution of seizure types in TUSZ v2.0.0 dataset.

Seizure Type	Number of Events	Total Duration (Seconds)
ABSZ	43	313.2
FNSZ	6924	396,670.1
SPSZ	942	31,985.1
TCSZ	247	11,869.4
TNSZ	380	7986.0

**Table 2:** Summary of Extracted Features per EEG Channel.

Domain	Features	Count
Temporal	Mean amplitude, standard deviation, maximum, minimum,	9
	interquartile range, median, variance, mean absolute amplitude, line length	
Frequency	Delta (0.5–4 Hz), Theta (4–8 Hz), Alpha (8–13 Hz),	5
	Beta (13–30 Hz), High Beta (30–40 Hz) power	
Total features per channel		14
Total features across 20 channels		280

**Table 3:** Detailed Architecture of EEG-ARCNet.

Layer	Maps	Size	Kernel/Stride/Pad	Parameters
*Input Stage*				
Input	1	20 × 14	-/-/-	0
*Initial Feature Extraction*				
Conv1D	64	20 × 64	7/1/same	6272
BatchNorm + ReLU	64	20 × 64	-/-/-	256
Channel Attention	64	20 × 64	-/-/-	1088
MaxPool1D	64	10 × 64	2/2/valid	0
*Feature Enhancement*				
Conv1D	128	10 × 128	5/1/same	41,088
BatchNorm + ReLU	128	10 × 128	-/-/-	512
Channel Attention	128	10 × 128	-/-/-	4224
Conv1D	128	10 × 128	5/1/same	82,048
BatchNorm	128	10 × 128	-/-/-	512
Skip Conv1D	128	10 × 128	1/1/same	8320
Add + ReLU	128	10 × 128	-/-/-	0
MaxPool1D	128	5 × 128	2/2/valid	0
*Deep Feature Processing*				
Conv1D	256	5 × 256	3/1/same	98,560
BatchNorm + ReLU	256	5 × 256	-/-/-	1024
Channel Attention	256	5 × 256	-/-/-	16,640
Conv1D	256	5 × 256	3/1/same	196,864
BatchNorm	256	5 × 256	-/-/-	1024
Skip Conv1D	256	5 × 256	1/1/same	33,024
Add + ReLU	256	5 × 256	-/-/-	0
*Classification Head*				
GlobalPool + Concat	512	512	-/-/-	0
Dense	512	512	-/-/-	262,656
BatchNorm + ReLU	512	512	-/-/-	2048
Dropout(0.5)	512	512	-/-/-	0
Dense (Softmax)	5	5	-/-/-	2565
Total Parameters:				758,725

**Table 4:** Computational requirements of EEG-ARCNet.

Metric	Value
**Model Complexity**	
Total Parameters	758,725
Model Size (FP32)	3.0 MB
**Computational Cost**	
FLOPs per Inference	15.2 M
Convolutional Operations	11.9 M (78%)
Attention Operations	1.2 M (8%)
Dense Layer Operations	2.1 M (14%)
**Memory Requirements**	
Parameter Memory	3.0 MB
Peak Activation Memory	8.5 MB
Runtime Memory (Pi 4B)	499.4 MB
RAM Utilization	6.2% (of 8 GB)

**Table 5:** Overall Classification Performance (Mean ± SD with 95% CI).

Metric	Performance (%)
Accuracy	99.65 ± 0.26 (99.32, 99.98)
Macro F1-Score	99.59 ± 0.28 (99.24, 99.94)
Macro Precision	99.33 ± 0.42 (98.81, 99.85)
Macro Recall	99.87 ± 0.08 (99.77, 99.97)

**Table 6:** Per-Class Performance Metrics (Mean ± SD across 5 folds).

Class	Sensitivity (%)	Specificity (%)	F1-Score (%)
ABSZ	100.00 ± 0.00	100.00 ± 0.00	100.00 ± 0.00
FNSZ	99.35 ± 0.51	100.00 ± 0.00	99.67 ± 0.26
SPSZ	100.00 ± 0.00	99.75 ± 0.29	98.84 ± 1.45
TCSZ	100.00 ± 0.00	99.98 ± 0.03	99.92 ± 0.10
TNSZ	100.00 ± 0.00	99.88 ± 0.11	99.53 ± 0.34

**Table 7:** Edge Device Performance Metrics on Raspberry Pi 4B.

Metric	Value
Average Inference Time	2.06 ms/segment
Maximum Inference Time	5.5 ms/segment
Real-Time Factor	4854×
CPU Utilization (Mean)	35.4%
CPU Utilization (Std Dev)	<1%
Memory Usage	499.4 MB
RAM Utilization	6.2% (of 8 GB)
Estimated Power Consumption	∼4.0 W
Battery Life (10,000 mAh)	∼9.25 h
Battery Life (20,000 mAh)	∼18.5 h
Classification Accuracy	99.2%

**Table 8:** Ablation Study: Performance and Computational Trade-offs.

Configuration	Accuracy (%)	F1-Score (%) *	Parameters
Baseline CNN	96.25	95.8	414,981
CNN + Attention Only	92.50	91.7	436,933
CNN + Residual Only	97.50	97.2	456,325
Full EEG-ARCNet	99.65	99.59	758,725

* F1-scores estimated from accuracy and class distribution.

**Table 9:** Attention Entropy Analysis Across Seizure Types.

Seizure Type	Entropy	Mean Weight	Interpretation
ABSZ	0.857 ± 0.004	0.075	Broadly distributed
FNSZ	0.847 ± 0.003	0.030	Broadly distributed
SPSZ	0.848 ± 0.004	0.035	Broadly distributed
TCSZ	0.852 ± 0.005	0.076	Broadly distributed
TNSZ	0.849 ± 0.006	0.096	Broadly distributed

**Table 10:** Performance Comparison with Recent Seizure Classification Approaches.

Study	Year	# Classes	F1-Score (%)
Liu et al. [14]	2020	8	97.4
Raghu et al. [13]	2020	8	88.3 ^‡^
Asif et al. [39]	2020	7	95.0
Priyasad et al. [32]	2021	8	96.7
Baghdadi et al. [22]	2022	8	98.41
Shankar et al. [49]	2022	6	98.8
Albaqami et al. [23]	2023	5	98.1
EEG-ARCNet	2025	5	99.59

^‡^ Accuracy reported; Note: Direct comparison is limited by differences in class numbers and dataset versions across studies.

**Table 11:** Computational Requirements Comparison with Recent Architectures.

Architecture	Parameters	FLOPs
Channel-wise Attention [32]	10.0M	850M
MP-SeizNet [23]	8.5M	620M
LSTM-Attention [22]	12.3M	1200M
EEG-ARCNet (Ours)	0.76M	15.2M
Reduction Factor	10–16×	40–79×

## Data Availability

The data presented in this study are available from the Temple University Hospital EEG Database at https://isip.piconepress.com/projects/nedc/html/tuh_eeg/ (accessed on 23 May 2024, registration required).

## Abbreviations

The following abbreviations are used in this manuscript:

ABSZ	absence seizure
ANN	artificial neural network
ASAP	acute seizure action plan
BiLSTM	bidirectional long short-term memory
CNN	convolutional neural network
CPU	central processing unit
DVFS	dynamic voltage and frequency scaling
EEG	electroencephalography
FLOP	floating-point operation
FNSZ	focal non-specific seizure
GPU	graphics processing unit
IQR	interquartile range
KNN	K-nearest neighbor
LSTM	long short-term memory
MFLOP	million floating-point operations
RAM	random access memory
ReLU	rectified linear unit
RF	random forest
SAP	seizure action plan
SPSZ	simple partial seizure
SUDEP	sudden unexpected death in epilepsy
SVM	support vector machine
TCSZ	tonic–clonic seizure
TCP-AR	TCP average reference
TNSZ	tonic seizure
TUH	Temple University Hospital
TUSZ	Temple University Hospital Seizure Corpus
VEM	video–EEG monitoring
WBAN	wireless body area network
